# Stem Cell-Based Therapies in Hearing Loss

**DOI:** 10.3389/fcell.2021.730042

**Published:** 2021-10-21

**Authors:** Zuhong He, Yanyan Ding, Yurong Mu, Xiaoxiang Xu, Weijia Kong, Renjie Chai, Xiong Chen

**Affiliations:** ^1^Department of Otorhinolaryngology-Head and Neck Surgery, Zhongnan Hospital of Wuhan University, Wuhan, China; ^2^Department of Otorhinolaryngology, Union Hospital, Tongji Medical College, Huazhong University of Science and Technology, Wuhan, China; ^3^State Key Laboratory of Bioelectronics, Jiangsu Province High-Tech Key Laboratory for Bio-Medical Research, School of Life Sciences and Technology, Southeast University, Nanjing, China; ^4^Co-Innovation Center of Neuroregeneration, Nantong University, Nantong, China; ^5^Institute for Stem Cell and Regeneration, Chinese Academy of Sciences, Beijing, China; ^6^Jiangsu Province High-Tech Key Laboratory for Bio-Medical Research, Southeast University, Nanjing, China; ^7^Beijing Key Laboratory of Neural Regeneration and Repair, Capital Medical University, Beijing, China

**Keywords:** stem cell, inner ear, hair cell, spiral ganglion neurons, hearing protection

## Abstract

In recent years, neural stem cell transplantation has received widespread attention as a new treatment method for supplementing specific cells damaged by disease, such as neurodegenerative diseases. A number of studies have proved that the transplantation of neural stem cells in multiple organs has an important therapeutic effect on activation and regeneration of cells, and restore damaged neurons. This article describes the methods for inducing the differentiation of endogenous and exogenous stem cells, the implantation operation and regulation of exogenous stem cells after implanted into the inner ear, and it elaborates the relevant signal pathways of stem cells in the inner ear, as well as the clinical application of various new materials. At present, stem cell therapy still has limitations, but the role of this technology in the treatment of hearing diseases has been widely recognized. With the development of related research, stem cell therapy will play a greater role in the treatment of diseases related to the inner ear.

## Introduction

Hearing disabilities have become one of the most common sensory disabilities in the world, but there is still no effective treatment for deafness (Wilson et al., 2017). Hearing loss can be classified as conductive hearing loss or SNHL according to the site of damage. The damage site for conductive hearing loss is mainly in the outer ear and middle ear, while the damage site for SNHL is mainly in the inner ear and auditory nerve (Weissman, 1996). At present, the treatment of SNHL mainly involves injections or oral drugs. In addition, local hormone injections, hyperbaric oxygen chamber rehabilitation, hearing aids, cochlear implantation, etc., can also be used in treatment (Chandrasekhar et al., 2019). The efficacy of treatment for patients in the acute phase is about 50%–70% (Tucci et al., 2002; Jeyakumar et al., 2006; Stachler et al., 2012). For patients who have not received effective treatment for more than 72 h after the onset of symptoms, the probability of hearing improvement will be greatly reduced. Some experts believe that the best time for initiating treatment should be within 48 h following the first aural symptoms (Ojha et al., 2020). However, even if the patient receives effective treatment in the acute phase, his hearing cannot be perfectly restored to the level before the illness (Stachler et al., 2012). Therefore, stem cell therapy may be an effective treatment for SNHL.

Inner ear hair cells and spiral ganglion neurons play a key role in the transmission of peripheral auditory signals (Nayagam et al., 2011; Moser and Starr, 2016). After exposure to the mechanical pressure of sound waves, the inner ear hair cells release neurotransmitters to the spiral ganglion cells, which then transmit signals to the auditory center. SNHL (SNHL) is caused by damage to the inner ear, auditory nerve, or central auditory pathway (Dufner-Almeida et al., 2019). The main factors that cause SNHL are damage to hair cells, damage to or loss of synapses between neurons and hair cells, and neuronal degeneration (Waqas et al., 2018). The loss of outer hair cells affects the function of the cochlear amplifier; the loss of inner hair cells or their synapses inhibits the encoding of sound signals; and the loss of spiral ganglia affects the encoding or conduction of sound signals (Moser et al., 2013). Therefore, the damage to the two kinds of inner ear nerve cells can cause permanent hearing loss (Lang, 2016). Previous studies have shown that non-mammalian vertebrates can regenerate hair cells in the cochlea and vestibular system after the hair cells are damaged to restore auditory function (Corwin and Cotanche, 1988). However, adult mammals have no regenerative ability for damaged hair cells, so hearing loss is permanent (Corwin and Cotanche, 1988; Brigande and Heller, 2009; Warchol, 2011). At present, the use of stem cells to induce differentiation to replace damaged hair cells is regarded as the most feasible treatment for regenerating hair cells. In addition, the loss of spiral ganglia, which are important to receiving incoming signals in the auditory system, is also irreversible. The loss of spiral neurons permanently damages the afferent pathways of auditory signals and causes SNHL (Shi and Edge, 2013). Therefore, implanting neural stem cells into the inner ear to regenerate spiral neurons and synaptic connections is also a potential way to restore hearing (Géléoc and Holt, 2014).

## The Role of Neural Stem Cells in Other Neurodegenerative Disease Treatment

Neural stem cells have strong proliferation and differentiation potential and can be specifically induced to differentiate into various nerve cells, such as neurons, astrocytes, and oligodendrocytes (Vieira et al., 2018). Therefore, neural stem cells are used as a potential solution for supplementing specific cells damaged by disease, such as neurodegenerative diseases, spinal injuries, and so on. Neural stem cells can be divided into autologous neural stem cells and allogeneic neural stem cells according to their sources. According to their different stages of growth and different tissue sources, neural stem cells can be divided into embryonic stem cell-derived neural stem cells, adult neural stem cells, and non-neural tissue-derived neural stem cells (Yi and Dong, 2010; Trounson and McDonald, 2015). At present, the therapeutic mechanisms of neural stem cells are mainly divided into three types: (1) neural stem cells gather at the injury site, proliferate, and differentiate into specific cells to restore the functions of the original tissues or organs; (2) neural stem cells secrete relevant nutritional factors to promote the recovery and regeneration of damaged cells; (3) neural stem cells establish or improve synaptic connections between neuronal cells and restore nerve conduction pathways.

A number of studies have reported that cell replacement therapy (CRT) using neural stem cells has made significant progress in neurodegenerative diseases such as Parkinson’s disease and Huntington’s disease (Choi and Hong, 2017; Marsh and Blurton-Jones, 2017). Generating specific neurons to function by implanting neural stem cells has become the focus of current research in the treatment of Parkinson’s disease. For example, newborn neurons are used to replace dopaminergic neurons in the striatum and participate in the reconstruction of the nervous system (Lindvall, 2015; Bjorklund and Parmar, 2020). Zhu et al. found that stem cells also have great potential in the treatment of amyotrophic lateral sclerosis (ALS) (Zhu and Lu, 2020). Implanted neural stem cells survive well in a damaged spinal cord. They not only replace lost motor neurons, but also act as a neuronal relay to establish connections between regenerating axons, and between their own axons and host axons so as to rebuild the body’s innervation of voluntary muscles (Zhu and Lu, 2020). The main pathological feature of Alzheimer’s disease (AD) is that amyloid β (Aβ) plaques accumulate in the degenerated neurons of the aging brain. Protein plaques are mainly composed of Aβ fibrils that phosphorylate tau protein and neurofibrillary tangles (NFTs). To treat AD, the implantation of neural stem cells restores damaged neurons, reduces Aβ accumulation, and ameliorates the microenvironment (Li et al., 2014; Han et al., 2020; Hayashi et al., 2020). Neural stem cell implantation also reduces brain damage in adult ischemic stroke and neonatal ischemic hypoxic encephalopathy through a variety of protective mechanisms such as immune regulation and neuroprotection. Endogenous neural stem cells can proliferate, differentiate, and repair brain damage under the stimulation of brain-derived neurotrophic factor (BDNF), NGF, EPO, etc. (Huang and Zhang, 2019). It is also reported that neural stem cell therapy is also used in the treatment of hemorrhagic encephalopathy (Gao et al., 2018), glioblastoma multiforme (Miska and Lesniak, 2015), multiple sclerosis (Xiao et al., 2018), and other diseases.

## The Role of Neural Stem Cells in Hearing Regeneration

During the embryonic development of mammals, as the expression of BMP changes, the non-neuroectoderm (NNE) at the junction of the neural tube and the ectoderm thickens, forming the pre-placodal ectoderm (PPE). Pre-placodal ectoderm forms the auditory placode at the front of the embryo. Under the induction of FGF (fibroblast growth factors) and Wnt released from the mesenchyme and neural tubes, the auditory placode is recessed and squeezed from the surface of the ectoderm to form an auditory vesicle. Then the SOX2-positive cell subset in the auditory vesicle up-regulates the pre-neural transcription factor bHLH and forms neuron precursor cells, which are separated from the auditory vesicle to form the cochlear-vestibular ganglion. The cells in the auditory vesicle form the sensory and non-sensory parts of the inner ear through proliferation, remodeling, and apoptosis (Roccio and Edge, 2019). The cochlear precursor cells in the organ of Corti have the ability to differentiate into neurospheres after birth (Zhai et al., 2005; Wang et al., 2006). Among these cells, Lgr5, Lgr6, Abcg2, EPCAM, and CD271 positive cells can proliferate and then differentiate into hair cells and supporting cells under the positive regulation of EGF (epidermal growth factor), IGF (insulin-like growth factor-1), bFGF (basic fibroblast growth factor), Wnt, Shh, and the negative regulation of p27Kip1. Atoh1, Shh, and the Notch pathways play an important regulatory role in the differentiation of precursor cells into hair cells. Nestin and Sox2-positive neural stem cells derived from spiral ganglia proliferate and differentiate into neurons and astrocytes under the control of EGF, IGF, bFGF, LIF (leukemia inhibitory factor), and other pathways (Xia et al., 2019). In this process, BDNF, *GDNF (glial cell-derived neurotrophic factor), NT-3 (neurotrophic factor-3), RA (valproic acid), FA (ferulic acid)* and other factors play an important regulatory role (Xia et al., 2019).

**FIGURE 1:**
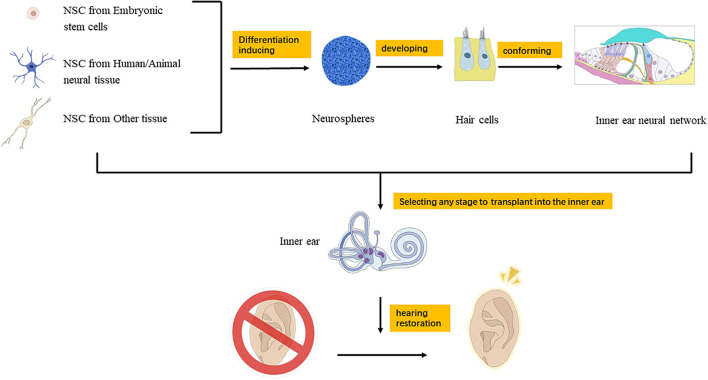
Mechanism of neural stem cell transplantation for the treatment of hearing loss.

In recent years, many scientists around the world have explored the application of neural stem cell therapy in the inner ear and have achieved many inspiring results. The main direction is to induce the regeneration of auditory hair cells and spiral ganglion cells to replace damaged cells and attempt to treat SNHL (Matsui et al., 2005; Nacher-Soler et al., 2019; Liu et al., 2020). Neural stem cells in the inner ear can differentiate into auditory neurons, hair cells, and supporting cells. Therefore, after the inner ear is damaged by noise, neural stem cells can make up for the damaged cells, meanwhile reduce the apoptosis of spiral ganglion cells (Xu et al., 2016). Iguchi et al. found that the effectiveness of cochlear implantation (CI) relies on residual spiral ganglion cells, and neural stem cells can differentiate into glial cells and neuronal cells after CI. GDNF and BDNF can nourish spiral ganglion cells to enhance hearing improvement after CI (Iguchi et al., 2003). The application of stem cell therapy in the inner ear mainly includes two aspects: stimulating the proliferation and differentiation of endogenous stem cells in the inner ear and implanting exogenous stem cells (Figure 1).

### Application of Endogenous Stem Cells in Hearing Regeneration

Studies have reported that there are inner ear stem cells in the cochlea and vestibule, which are distributed in the greater epithelial ridge (GER), lesser epithelial ridge (LER), organ of Corti, vestibular sensory epithelium, and semicircular canals (Liu et al., 2014). The inner ear stem cells in the mouse cochlea can be isolated in the first week after birth, while the stem cells in the vestibule can be isolated even 4 months after birth (Oshima et al., 2007; Kanzaki et al., 2020). Inner ear stem cells are regulated by a variety of transcription factors and can differentiate into sensory precursor cells, neural precursor cells, and non-sensory cells (Kiernan et al., 2005; Raft et al., 2007). Genes such as *Jagged1* (Daudet and Lewis, 2005), *Notch1* (Liu et al., 2012), *Sox2* (Neves et al., 2007), *BMP-4* (Cole et al., 2000), *FGF* (Schimmang, 2007), *IGF-1* (Aburto et al., 2012), *Atoh1*, *Jagged2*, and *Delta1* (Morrison et al., 1999) play important regulatory roles in the differentiation and development of inner ear stem cells into hair cells. In addition, *Brn3c* (Xiang et al., 1997), *Espin* (Zheng et al., 2000), and *Myosin VI*, *VIIA*, and *XV* (Steel and Kros, 2001; Udovichenko et al., 2002) are important for the survival of hair cells, and *TGF-*α promotes the transdifferentiation of supporting cells into hair cells (Liu et al., 2014). Neural stem cells in the inner ear also have the potential to replace damaged cells, and these neural stem cells may be derived from residual spiral ganglion cells (Oshima et al., 2007, 2009). Previous audiology-related studies have found that the number of remaining spiral ganglion neurons has an effect on speech recognition after CI (Seyyedi et al., 2014).

The research on the differentiation of inner ear precursor cells (such as stem cells or supporting cells) into hair cells was first carried out in non-mammalians. Researchers found that after the inner ear hair cells of non-mammals such as birds, fish, and amphibians are damaged, the supporting cells directly or indirectly transdifferentiate into hair cells (Bodson et al., 2010; Wang et al., 2015; Kanzaki, 2018). There are two ways to regenerate hair cells from inner ear supporting cells: re-entering the cell cycle, and transdifferentiation (Chen et al., 2019). In addition, Lagarde et al. found that when the organ of Corti in newborn mice is not fully mature, two types of supporting cells, inner border cells and inner finger cells, can be effectively replenished after loss, thereby maintaining normal hearing in mice (Mellado Lagarde et al., 2014). Cox et al. found that when the cochlear hair cells of newborn mice are lost, supporting cells can regenerate hair cells through mitosis and transdifferentiation, although most of the regenerated hair cells are gradually lost with an extension of development time (Cox et al., 2014). These prove that when the cochlea of newborn mice is damaged, it can activate its ability to regenerate hair cells. It is known that the current technical methods for inducing the regeneration of supporting cells into hair cells mainly include gene editing and drug treatment (Géléoc and Holt, 2014). In 2005, Izumikawa et al. used adenoviral vectors to transfect the *Atoh1* gene into the inner ear for the first time. *Atoh1* can achieve partial hearing recovery and improvement after deafness by encoding HLH transcription factors and the key factors related to hair cell development (Izumikawa et al., 2005). Akil et al. used adeno-associated virus type 1 (AAV1) to deliver the *VGLUT3* gene to the inner ears of *VGLUT3* knockout mice and found that the morphology of the ribbon synapses between the inner hair cells was restored. Within 2 weeks, the examined result of mouse auditory brainstem response (ABR) threshold returned to normal level, and the startle reflex was partially relieved (Akil et al., 2012). At present, the application of genetic engineering in the treatment of deafness still has many limitations. For example, the research of Masahiko Izumikawa et al. failed to restore hearing in all experimental animals (Izumikawa et al., 2005). The *VGLUT3* mutation studied by Akil et al. is also not common in humans, so it does not have broad representative significance (Akil et al., 2012). However, the value and potential of therapy through the gene introduction of viral vectors have been reflected in many studies. In addition, other gene therapy methods such as the introduction of siRNA, knockout of dominant genes, systemic injection of antisense oligonucleotides, and plasmid introduction into intrauterine embryos also show good therapeutic effects and can be used as potential therapeutic methods (Muller and Barr-Gillespie, 2015). It has been confirmed that some genes in the signaling pathways related to the regeneration of inner ear hair cells play important regulatory roles, such as *Atoh1* (Bermingham et al., 1999; Chonko et al., 2013), *p27Kip1* (Chai et al., 2011), *pRb* (Sage et al., 2006), *Foxg1* (Ding et al., 2020), *Wnt* (Bengoa-Vergniory and Kypta, 2015), *Notch* (Kiernan, 2013), *Hedgehog* (Zhao et al., 2006), *Ephrin*, *Six1*, *Pou4f3*, and *Gfi1* (Menendez et al., 2020; Zhang et al., 2020a). White et al. found that down-regulating the expression of the cell cycle inhibitor *P27Kip1* enabled some of the supporting cells in the inner ear to re-enter the cell cycle and generate hair cells (White et al., 2006). Mizutari et al. injected γ-secretase inhibitors locally in mice with noise-induced hearing loss to inhibit the expression of Notch and increase the level of Atoh1. They found that the transdifferentiation of supporting cells into hair cells occurred in the inner ears of mice, resulting in an increase in the number of hair cells (Mizutari et al., 2013). Menendez et al. combined the four transcription factors Six1, Atoh1, Pou4f3, and Gfi1 to convert mouse embryonic fibroblasts, adult mouse tail fibroblasts and postnatal mouse supporting cells into induced hair cell-like cells (Menendez et al., 2020). Foxg1 can affect the proliferation of inner ear neural progenitor cells by regulating the expression of genes related to the cell cycle and Notch signaling pathway. Zhang et al. found that knockout *Foxg1* can promote the transdifferentiation of supporting cells to hair cells (Zhang et al., 2020b). Sage et al. found that pRb plays an important role in the maturation and survival of auditory hair cells. When the expression of pRb is deleted, the vestibular hair cells and supporting cells of postnatal mice still divide and proliferate (Sage et al., 2006). Although the hair cells regenerated in this way cannot fully restore the number of cells before the injury, and the hearing improvement is limited (only about 10 dB), this study confirmed the feasibility of regenerating hair cells through the regulation of the cell cycle by drugs, and also promoted the application of more cell cycle regulators in the future (Mizutari et al., 2013; Géléoc and Holt, 2014; Kanzaki et al., 2020).

Recent studies have shown that microRNA is also a potential gene therapy tool. It not only affects the development of the cochlea and hair cells, but also regulates the proliferation and differentiation of inner ear stem cells, which is very important for the regeneration of inner ear hair cells (Wu et al., 2020). Jiang et al. found that regulating the expression of miR-124 in inner ear neural stem cells in spiral ganglia can change the expression of tropomyosin receptor kinase B (TrkB) and cell division cycle 42 (Cdc42), and it promotes the neuronal differentiation and neurite outgrowth of inner ear neural stem cells (Jiang et al., 2016). At present, many studies have tried to use the regulatory role of microRNA in cell proliferation and differentiation to repair and regenerate inner ear hair cells, thereby treating hearing loss (Chen et al., 2018; Zhou et al., 2018).

### Application of Exogenous Stem Cells in Hearing Regeneration

Due to the limited number of existing stem cells in the inner ear, and because the mechanism of inner ear cell renewal is still unclear, many researchers have tried to repair inner ear cells by implanting neural stem cells (Waqas et al., 2020). Clarke et al. found that neural stem cells have the potential to differentiate into functional auditory neurons (Clarke et al., 2000). The reported sources of neural stem cells implanted in the inner ear include dorsal root ganglion cells, neural precursor cells, the stem cells or precursor cells isolated from the inner ear, immortalized auditory neuroblasts, embryonic stem cells and their derived neural stem cells, and bone marrow stromal cells treated with Shh and retinoic acid (Lang et al., 2008). Michael et al. developed an organoid culture system *in vitro* based on the *in vivo* embryonic development system (Perny et al., 2017). They first activated BMP and inhibited TGF-β to induce mouse embryonic stem cells (mESCs) to generate non-neuroectoderm, while avoiding the induction of mesoderm, and then inhibited BMP and activated FGF2 to further induce the generation of pre-placodal ectode (PPE) and otic placode. Spiral ganglia were stratified and differentiated in a serum-free 2D Matrigel matrix. The tissues were treated with BDNF and NT-3 for 15 days *in vitro*, and were finally differentiated into mature spiral ganglia with a clear morphology and normal function (Perny et al., 2017). Karl R. Koehler et al. used the quickly aggregated serum-free embryonic body method (SFEBq) to culture mouse embryonic neural stem cells, and regulated the expression of BMP, TGF-β, and FGF at different time points, so that the cell population formed non-neuroectoderm, PPE, and otic placode epithelial cells. The signal pathways related to the differentiation of the inner ear sensory epithelial cells were then are activated, such as the Wnt, Notch, Hippo, Shh, and MAPK pathways (Bengoa-Vergniory and Kypta, 2015; Ouyang et al., 2020; Susanto et al., 2020), resulting in a large number of hair cells with special function and structure that could sense mechanical pressure (Koehler et al., 2013; Jiao et al., 2017; Xia et al., 2019). In addition, nerve growth factor (NGF) plays an important role in the survival and differentiation of neural stem cells. A medium containing NGF has a large number of neural stem cells with high differentiation potential (Han et al., 2017).

## Method and Function Evaluation of Neural Stem Cell Implantation in the Inner Ear

Implanting stem cells into the inner ear can select proper pathway from perilymph, endolymph, cochlear axis, auditory nerve, cochlear lateral wall, and so on (Zhu et al., 2018). The perilymph path includes round window and external semicircular canal injection, and the endolymph path is through membranous cochlear duct injection (Liu et al., 2016). Zhang et al. cultivated neural stem cells for a period of time, and then injected them into the cochlea through the cochlear sidewall, allowing them to migrate to the area of the cochlea axis where the spiral ganglia were distributed (Zhang et al., 2013). This method is effective, precise, and incurs a minimal level of trauma. Due to the special structure of the cochlea, invasive cochlear surgery may cause severe hearing loss (Bogaerts et al., 2008). Therefore, when neural stem cells are implanted, different methods should be selected according to the treatment conditions and treatment purposes (Figure 1).

It is necessary to test the function of neural stem cells after implantation from the perspective of histology and function. Histological detection indicators mainly include the differentiation of neural stem cells, the neurotrophic factors secreted by neural stem cells, and the formation of neural networks such as the extension of axons and the establishment of synaptic connections between neurons. Functional detection indicators mainly include the improvement in the hearing level of the implanted object, whether symptoms such as tinnitus are alleviated, and whether the effect of hearing devices such as cochlear implants has been enhanced. To determine whether neural stem cells are successfully differentiated into target cells after implantation in the inner ear, detection is mainly based on morphology, protein expression, and genetic markers. For example, detection may be based on detecting specific expression genes (*MYO7A*, *BRN3A*, and *ATHO1*), auditory receptors, mechanical energy to electrical energy conversion, and hair cell electrophysiological activity to determine whether the newly generated hair cells after stem cell implantation have the characteristics of normal hair cells (Ottersen et al., 1998; Gale et al., 2001; Griesinger et al., 2002; Prosser et al., 2008; Beurg et al., 2009). The BrdU detection of cell proliferation, microscopic detection of morphology, and detection of synaptic protein expression, as well as electrophysiological detection and other methods can determine whether the implanted newly generated cells have successfully differentiated into spiral ganglion cells (Li et al., 2016).

## Application of New Materials Related to Neural Stem Cells in the Treatment of Auditory Diseases

**FIGURE 2:**
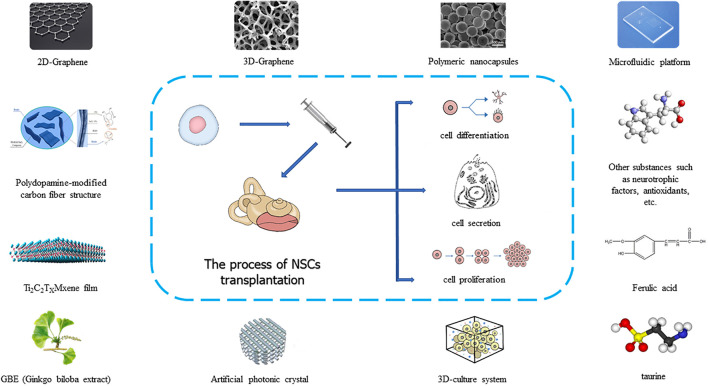
Application of new materials and new substrates in neural stem cell transplantation.

In recent years, many researchers have developed more new technologies and materials in the process of using neural stem cells to treat auditory diseases, and these technologies have promoted the clinical application of neural stem cells (Figure 2). As a material with excellent stability, biocompatibility, conductivity, ductility, elasticity, and mechanical strength, graphene is often used in tissue engineering research. When graphene was used as a nanocomposite carrier or scaffold material for neural stem cells, researchers found that graphene materials could promote the proliferation and differentiation of neural stem cells and the directional growth of neuronal axons, and ultimately formed biologically functional tissue (Shin et al., 2016; Yang et al., 2018; Han et al., 2019). When neural stem cells were cultured on a graphene substrate, the cell membrane potential parameters did not change, but when neural stem cells proliferated and differentiated, the resting potential of the cells increased negatively, and the amplitude of the action potential increased. In addition, the differentiation of neural stem cells accelerated, and the expression of synaptic proteins and synaptic activity increased, which showed that graphene could accelerate the development and maturation of neural stem cells (Guo et al., 2016). In addition to graphene, artificial photonic crystal materials also promote the growth of neural stem cells due to their special topological properties and electrical signal stimulation (Yang et al., 2013; Ankam et al., 2015). Besides these new materials, anisotropic inverse opal is a material that regulates the behavior of neural stem cells by changing their surface morphology. Compared with isotropic inverse opal, special 3D (3-dimensional) porous structure of anisotropic inverse opal can make neural stem cell spheres have stronger proliferation ability, more orderly cell arrangement, better directional differentiation, and a significantly higher dendritic complexity index (DCI) (Xia et al., 2020). The use of a 3D culture system can simulate the inner ear microenvironment and promote the complete formation of stem cells into a functional structure of the inner ear (Chang et al., 2020). When neural stem cells are implanted in the inner ear to treat auditory diseases, different materials can be selected according to different treatment requirements (Figure 2). To date, extensive research has been carried out on the main processes of neural stem cell acquisition, implantation, and postoperative inner ear functional recovery. However, there are still unresolved problems related to tumorigenicity, targeted growth, and cell survival rate after implantation. Therefore, more precise and effective optimization of treatment methods is needed in the future.

## Conclusion

At present, great progress has been made in the research on endogenous and exogenous neural stem cells in the treatment of auditory diseases. A large number of studies have covered the acquisition, induction, and implantation of neural stem cells, and the restoration of auditory function after implantation. Neural stem cells are implanted into the inner ear to replace and supplement hair cells or spiral ganglion cells, to promote the renewal and proliferation of residual cells and to restore or rebuild the neuron network, so as to achieve the recovery of auditory function (Figure 1). This is a valuable and promising treatment method for auditory diseases. However, there are still unknown factors in the inner ear implantation of neural stem cells, such as tumorigenicity and immune rejection. Moreover, functional recovery after implantation has not reached a satisfactory level for clinical application. In the future, research on inner ear stem cells will discover new materials and regulatory genes or proteins, which will promote the clinical application of neural stem cells.

## Author Contributions

ZH, YD, YM, and XX searched and read related literature, summarized the data in the field, and wrote the manuscript. ZH, RC, and XC guided the writing and review of the manuscript. All authors contributed to the article and approved the submitted version.

## Conflict of Interest

The authors declare that the research was conducted in the absence of any commercial or financial relationships that could be construed as a potential conflict of interest.

## Publisher’s Note

All claims expressed in this article are solely those of the authors and do not necessarily represent those of their affiliated organizations, or those of the publisher, the editors and the reviewers. Any product that may be evaluated in this article, or claim that may be made by its manufacturer, is not guaranteed or endorsed by the publisher.
